# Supplementary material for: Side-Arm Assisted Anilido-Imine Based Rare-Earth Metal Complexes for Isoprene Stereoselective Polymerization
Source: Molecules. 2021 Jul 8;26(14):4154. doi: 10.3390/molecules26144154 (PMC8306391; doi:10.3390/molecules26144154)
Supplement: Supplementary file 1 [file molecules-26-04154-s001.zip › molecules-1272433-supplementary.pdf]

Synthesis of Rare-Earth Metal Complexes with Chelating Anilido-Imine Ligands and Catalysis for Isoprene Polymerization

Yi Wu [1,2], *Xinli Liu* [1,2,*] *and Dongmei Cui* [1,2,*]

[1]State Key Laboratory of Polymer Physics and Chemistry, Changchun Institute of Applied Chemistry, Chinese Academy of Sciences, Changchun 130022, China.

[2]Department of Polymer Science and Engineering, University of Science and Technology of China. Hefei 230026, China.

Contents

Figure S1. ^{1}H NMR spectrum (500 MHz, $CDCl_3$, 25 °C) of ligand HL_1.

Figure S2. ^{1}H NMR spectrum (500 MHz, $CDCl_3$, 25 °C) of ligand HL_2.

Figure S3. ^{1}H NMR spectrum (500 MHz, C_6D_6, 25 °C) of complex **1a**.

Figure S4. ^{13}C NMR spectrum (125 MHz, C_6D_6, 25 °C) of complex **1a**.

Figure S5. ^{1}H NMR spectrum (500 MHz, C_6D_6, 25 °C) of complex **1b**.

Figure S6. ^{13}C NMR spectrum (125 MHz, C_6D_6, 25 °C) of complex **1b**.

Figure S7. ^{1}H NMR spectrum (500 MHz, C_6D_6, 25 °C) of complex **1c**.

Figure S8. ^{13}C NMR spectrum (125 MHz, C_6D_6, 25 °C) of complex **1c**.

Figure S9. ^{1}H NMR spectrum (500 MHz, C_6D_6, 25 °C) of complex **2a**.

Figure S10. ^{13}C NMR spectrum (125 MHz, C_6D_6, 25 °C) of complex **2a**.

Figure S11. ^{1}H NMR spectrum (500 MHz, C_6D_6, 25 °C) of complex **2b**.

Figure S12. ^{13}C NMR spectrum (125 MHz, C_6D_6, 25 °C) of complex **2b**.

Figure S13. ^{1}H NMR spectrum (500 MHz, C_6D_6, 25 °C) of complex **2c**.

Figure S14. ^{13}C NMR spectrum (125 MHz, C_6D_6, 25 °C) of complex **2c**.

Figure S15. ^{1}H NMR spectrum (500 MHz, $CDCl_3$, 25 °C) of *cis*-1,4 PIP (Table 1, entry 3).

Figure S16. ^{13}C NMR spectrum (125 MHz, $CDCl_3$, 25 °C) of *cis*-1,4 PIP (Table 1, entry 3).

Figure S17. ^{1}H NMR spectrum (500 MHz, $CDCl_3$, 25 °C) of *trans*-1,4 PIP (Table 1, entry 4).

Figure S18. ^{13}C NMR spectrum (125 MHz, $CDCl_3$, 25 °C) of *trans*-1,4 PIP (Table 1, entry 4).

Table S1. Crystal data and structure refinement for complexes **1a**, **1c, 2a, 2c**.

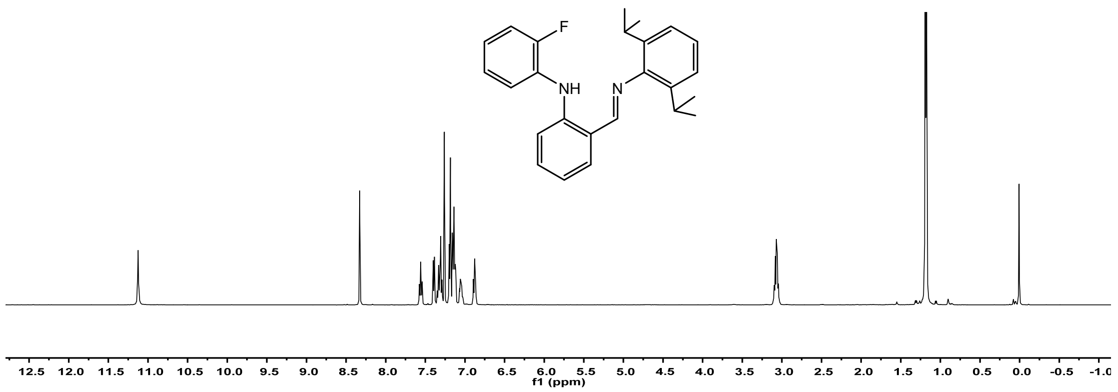

Figure S1. ^{1}H NMR spectrum (500 MHz, $CDCl_3$, 25 °C) of ligand HL_1.

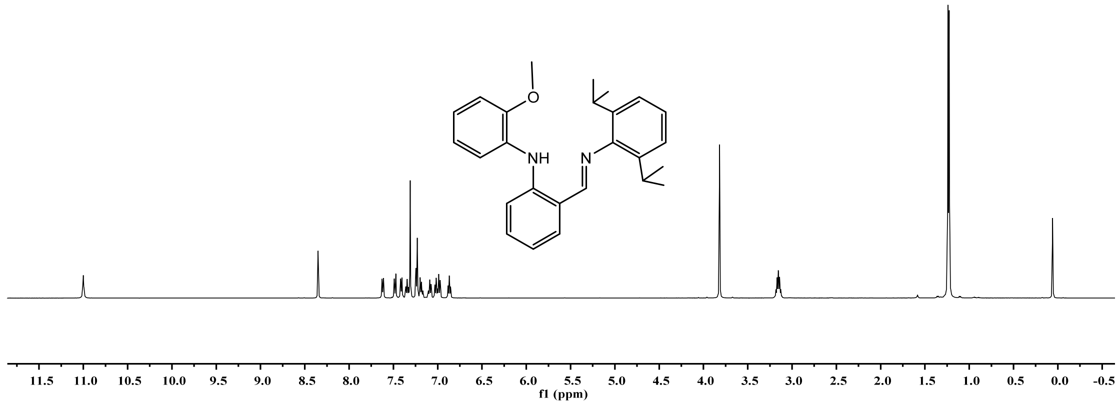

Figure S2. ^{1}H NMR spectrum (500 MHz, $CDCl_3$, 25 °C) of ligand HL_2.

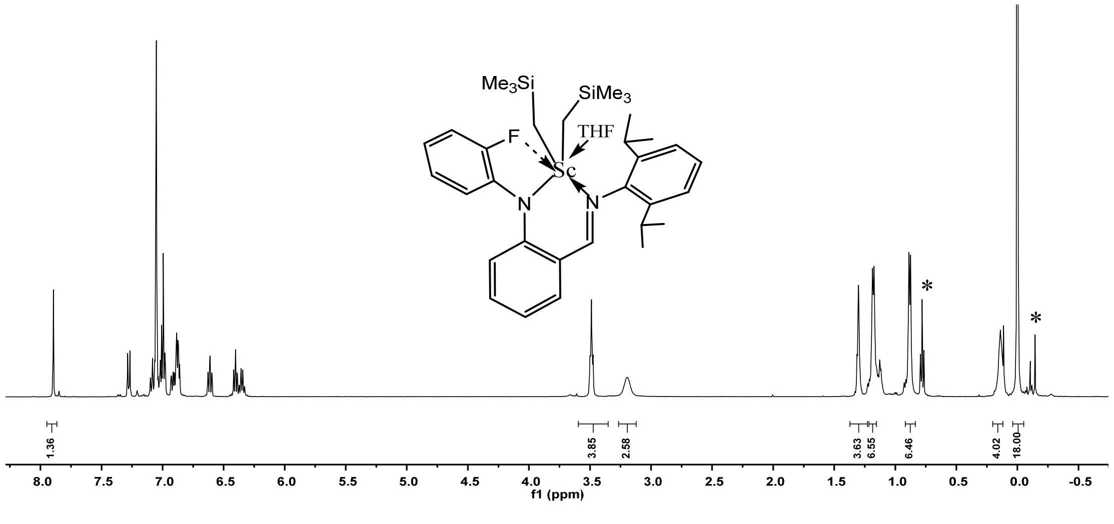

Figure S3. ^{1}H NMR spectrum (500 MHz, C_6D_6, 25 °C, *: impurity) of complex **1a**.

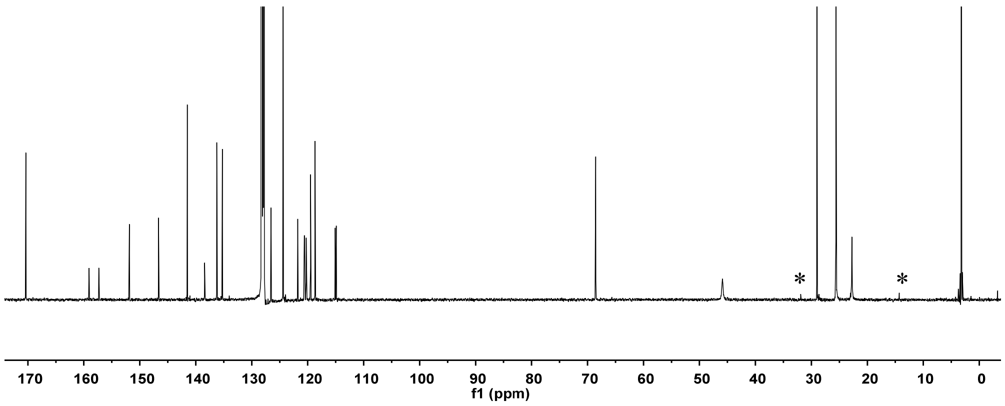

Figure S4. ^{13}C NMR spectrum (125 MHz, C_6D_6, 25 °C, *: impurity) of complex **1a**.

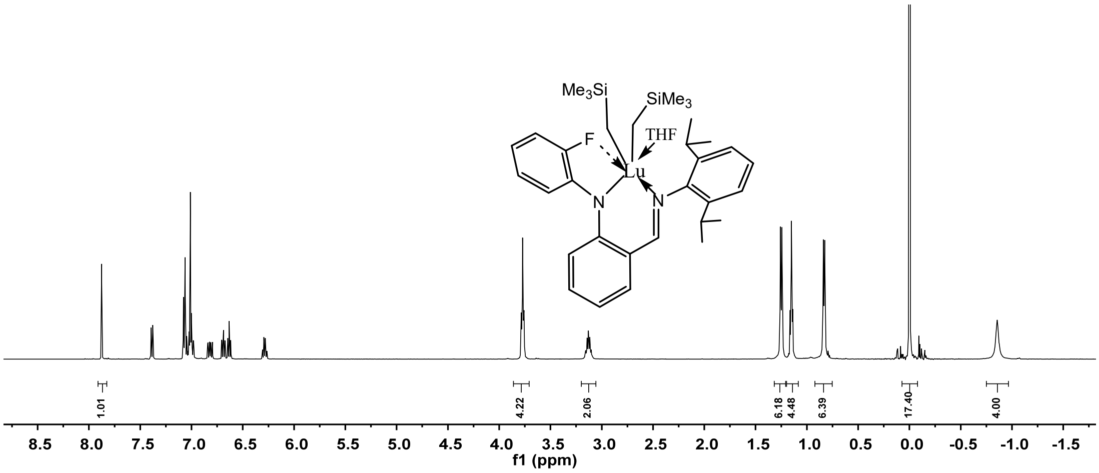

Figure S5. ^{1}H NMR spectrum (500 MHz, C_6D_6, 25 °C) of complex **1b**.

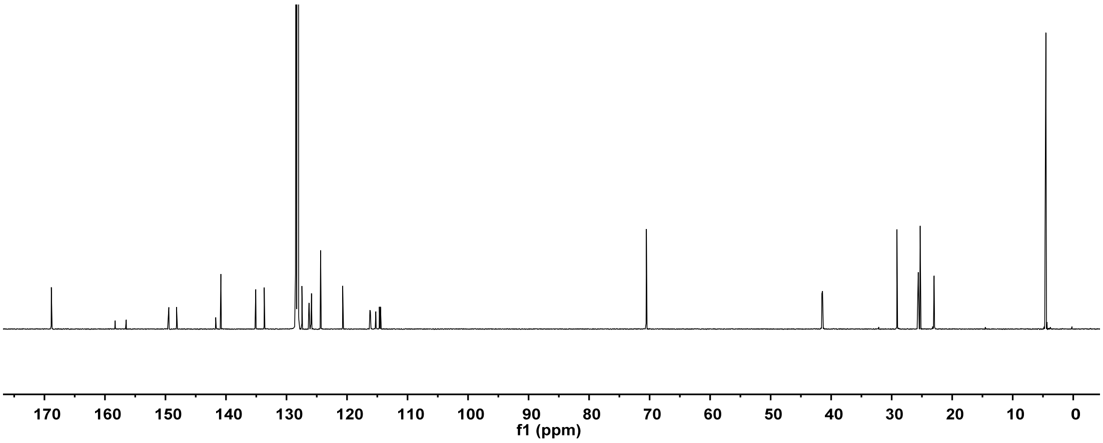

Figure S6. ^{13}C NMR spectrum (125 MHz, C_6D_6, 25 °C) of complex **1b**.

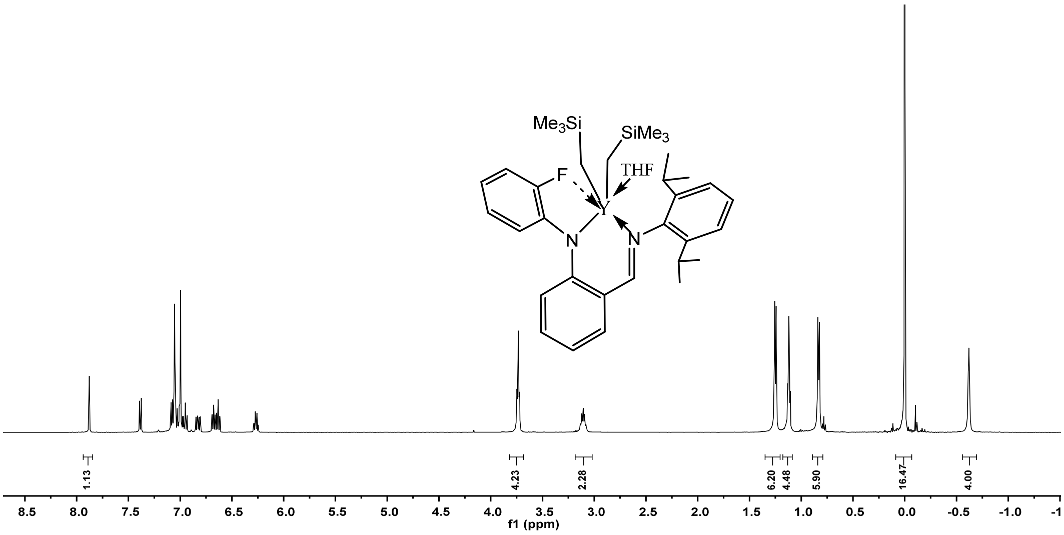

Figure S7. ^{1}H NMR spectrum (500 MHz, C_6D_6, 25 °C) of complex **1c**.

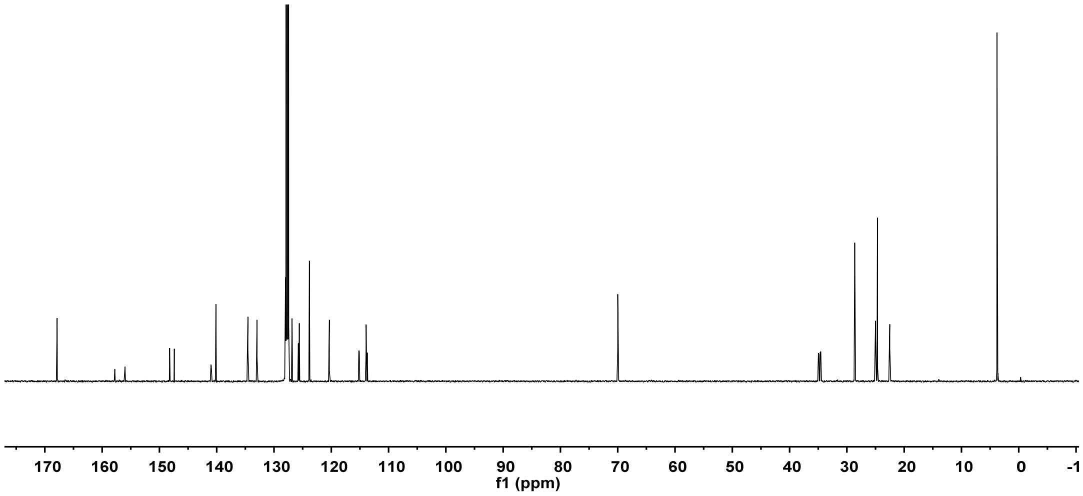

Figure S8. ^{13}C NMR spectrum (125 MHz, C_6D_6, 25 °C) of complex **1c**.

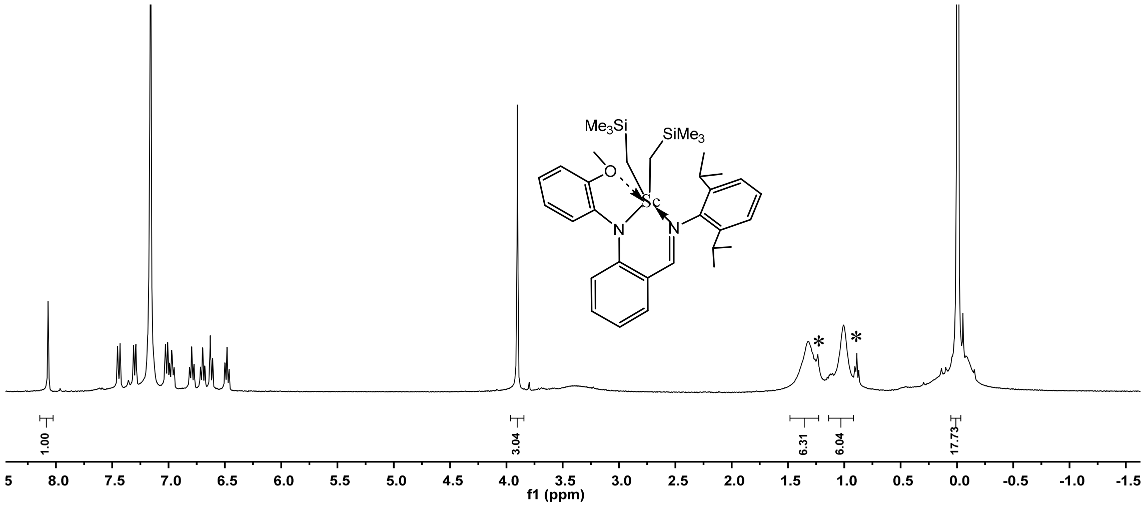

Figure S9. ^{1}H NMR spectrum (500 MHz, C_6D_6, 25 °C, *: n-hexane) of complex **2a**.

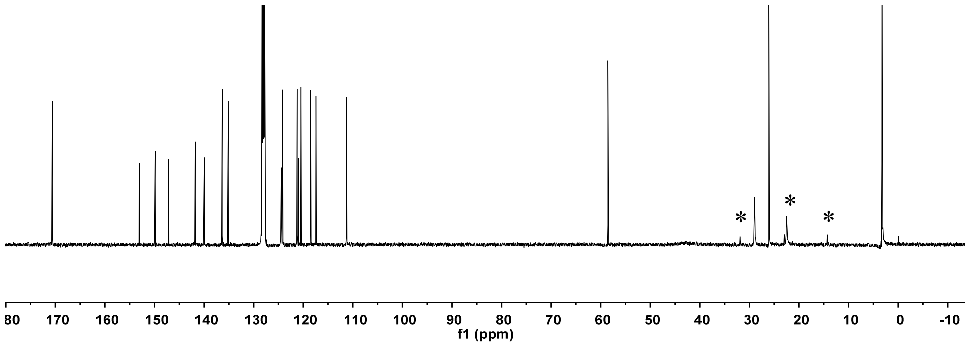

Figure S10. ^{13}C NMR spectrum (125 MHz, C_6D_6, 25 °C,*: n-hexane) of complex **2a**.

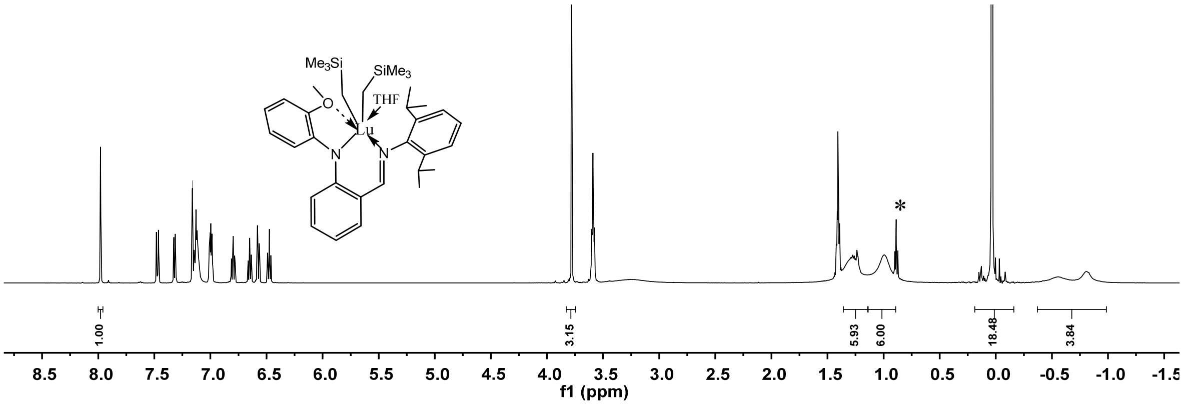

Figure S11. ^{1}H NMR spectrum (500 MHz, C_6D_6, 25 °C, *: n-hexane) of complex **2b**.

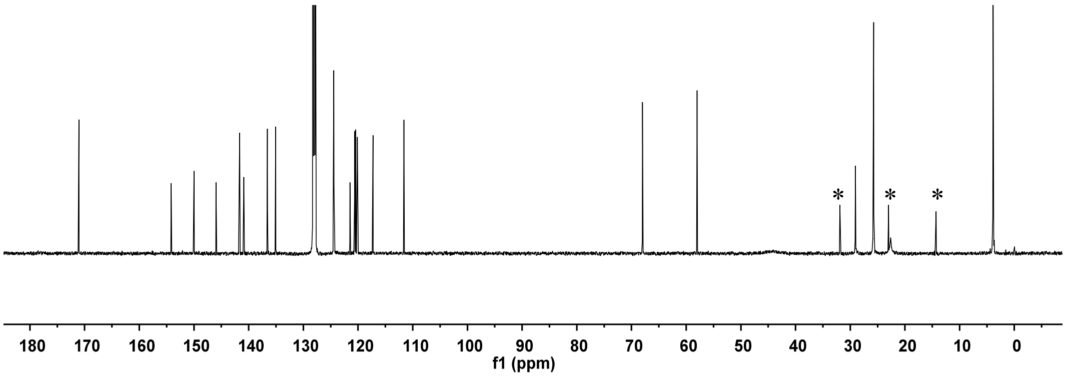

Figure S12. ^{13}C NMR spectrum (125 MHz, C_6D_6, 25 °C, *: n-hexane) of complex **2b**.

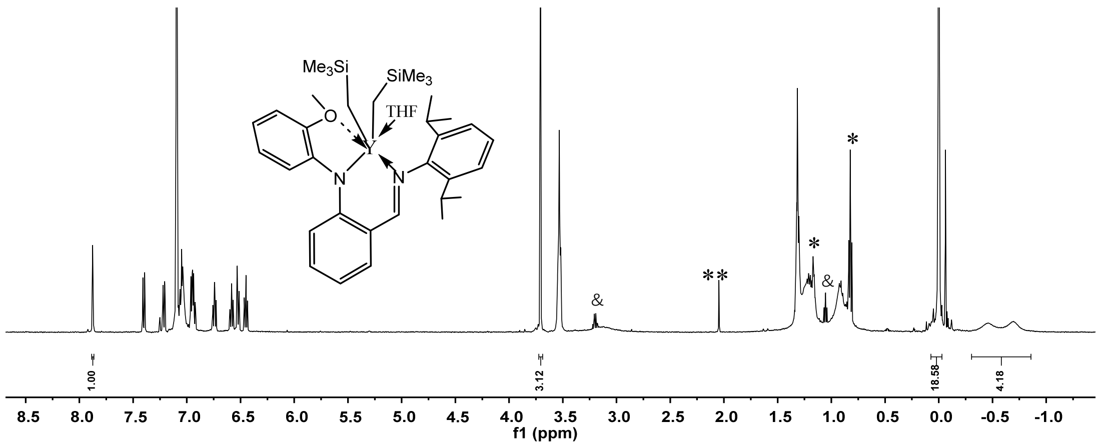

Figure S13. ^{1}H NMR spectrum (500 MHz, C_6D_6, 25 °C, *: n-hexane, **: toluene, &: impurity) of complex **2c**.

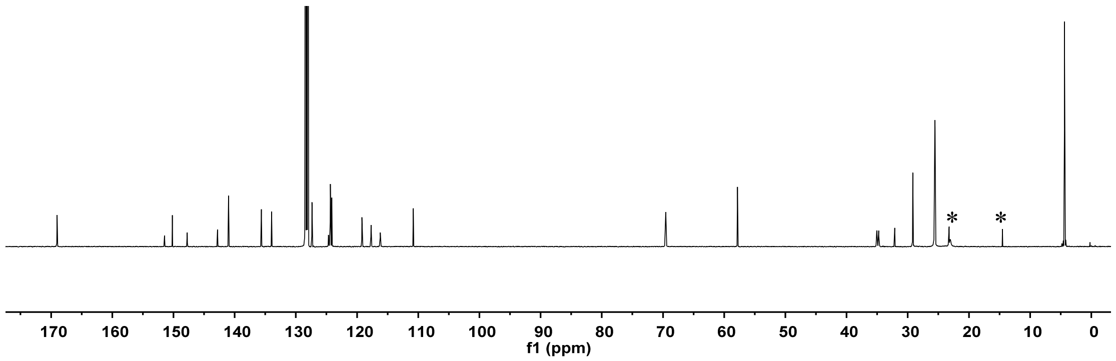

Figure S14. ^{13}C NMR spectrum (125 MHz, C_6D_6, 25 °C, *: n-hexane) of complex **2c**.

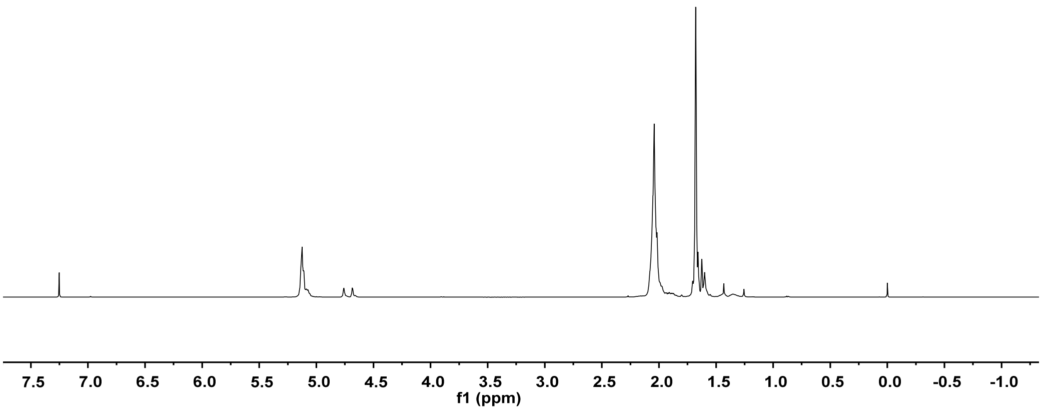

Figure S15. ^{1}H NMR spectrum (500 MHz, $CDCl_3$, 25 °C) of *cis*-1,4 PIP (Table 1, entry 3).

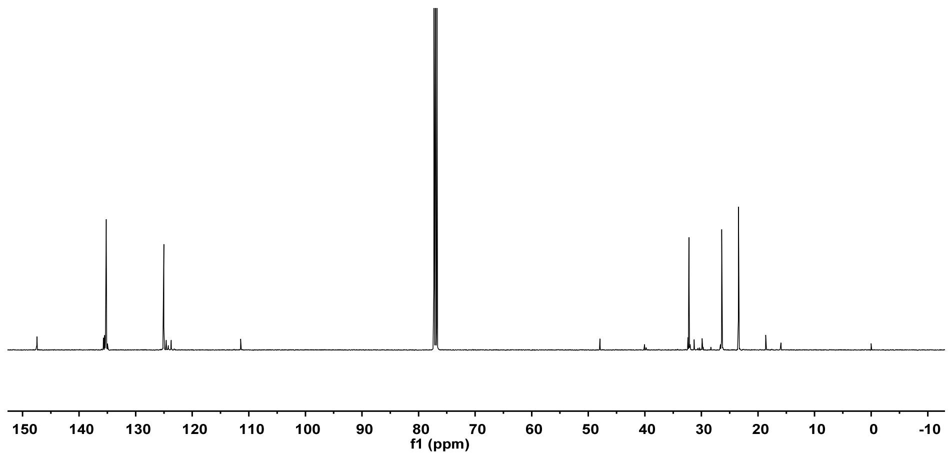

Figure S16. ^{13}C NMR spectrum (125 MHz, $CDCl_3$, 25 °C) of *cis*-1,4 PIP (Table 1, entry 3).

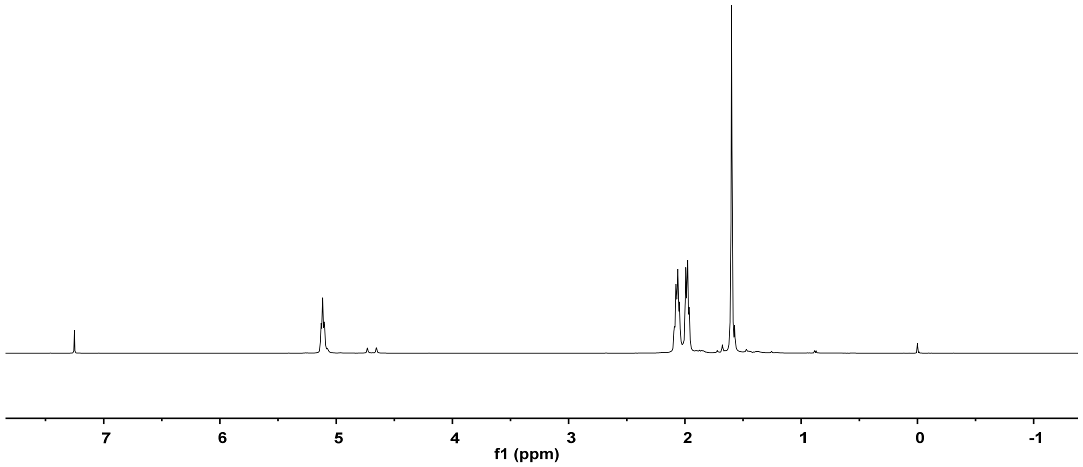

Figure S17. ^{1}H NMR spectrum (500 MHz, $CDCl_3$, 25 °C) of *trans*-1,4 PIP (Table 1, entry 4).

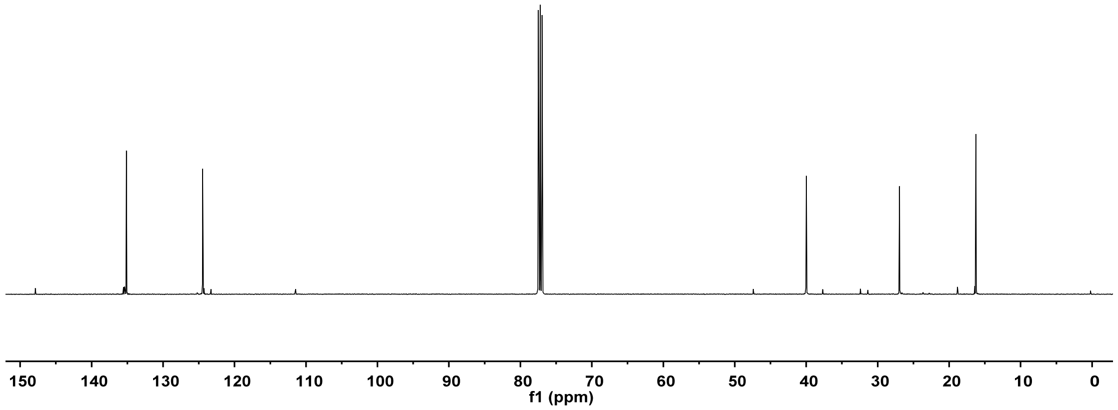

Figure S18. ^{13}C NMR spectrum (125 MHz, $CDCl_3$, 25 °C) of *trans*-1,4 PIP (Table 1, entry 4).

Table S1. Crystal data and structure refinement for complexes **1a**, **1c, 2a**, **2c**.

Identification code	**1a**	**1c**	**2a**	**2c**
Formula	$C_{37}H_{55}FN_2OSi_2Sc$	$C_{37}H_{56}FN_2OSi_2Y$	$C_{34}H_{51}N_2OScSi_2$	$C_{38}H_{59}N_2O_2Si_2Y$
Formula weight	664.47	708.92	604.93	716.93
Crystal dimensions (mm^3)	0.2×0.18×0.15	0.3×0.2×0.1	0.3×0.1×0.1	0.3×0.1×0.1
Crystal system	monoclinic	orthorhombic	triclinic	monoclinic
Space group	P 1 21/c 1	P 21 21 21	P -1	P 1 21/n 1
a(Å)	10.5934(13)	12.4186(7)	10.1127(3)	11.3439(4)
b(Å)	38.048(5)	13.5705(7)	10.5747(4)	19.8521(7)
c(Å)	19.364(2)	24.0659(13)	17.8379(6)	18.1172(7)
α(°)	90	90	92.8500(10)	90
β(°)	93.539(3)	90	91.9980(10)	97.7780(10)
γ(°)	90	90	110.4620(10)	90
Volume (Å^3)	7790.0(16)	4055.7(4)	1782.21(11)	4042.5(3)
Z	4	4	1	4
T (K)	273(2)	273(2)	273(2)	273(2)
D_{calcd} (g cm^{-3})	1.133	1.161	1.1272	1.178
μ (mm^{-1})	0.28	1.53	0.300	1.533
F (000)	2860	1504	653.0590	1520
Rflns.collected	45057	23187	65918	71899
Indep.rflns./R_{int}	15784/0.1413	8170/0.0625	5593/0.0611	11780/0.0722
Obsd.rflns.[I_0>2σ(I_0)]	6770	5615	4836	7764

Data/restraints/parameters	15784/0/813	8170/6/395	5593/0/372	11780/0/418
R_1 / wR_2 [I_0 > 2σ(I_0)]	0.0777/0.1335	0.0549/0.1245	0.0336/0.0772	0.0519/0.1135
R_1 / wR_2 (all data)	0.1981/0.1776	0.0894/0.1394	0.0425/0.0850	0.0954/0.1298
GOF (on F^2)	0.952	0.969	1.0888	1.026
dihedral angel	49.625	53.3	44.392	50.21
Largest diff. peak and hole (e Å−3)	0.369/-0.308	0.870/-0.578	0.2358/-0.3123	1.060/-0.567
